# Phage and Nucleocytoplasmic Large Viral Sequences Dominate Coral Viromes from the Arabian Gulf

**DOI:** 10.3389/fmicb.2017.02063

**Published:** 2017-10-24

**Authors:** Huda Mahmoud, Liny Jose

**Affiliations:** Faculty of Science, Department of Biological Sciences, Kuwait University, Safat, Kuwait

**Keywords:** viral metagenomes, *Acropora downingi*, *Porites harrisoni*, extreme conditions, Arabian Gulf

## Abstract

Corals that naturally thrive under extreme conditions are gaining increasing attention due to their importance as living models to understand the impact of global warming on world corals. Here, we present the first metagenomic study of viral communities in corals thriving in a thermally variable water body in which the temperature fluctuates between 11 and 39°C in different seasons. The viral assemblages of two of the most abundant massive (*Porites harrisoni*) and branching (*Acropora downingi*) corals in offshore and inshore reef systems in the northern Arabian Gulf were investigated. Samples were collected from five reef systems during summer, autumn and winter of 2011/2012. The two coral viromes contain 12 viral families, including 10 dsDNA viral families [Siphoviridae, Podoviridae, Myoviridae, Phycodnaviridae, Baculoviridae, Herpesviridae, Adenoviridae, Alloherpesviridae, Mimiviridae and one unclassified family], one-ssDNA viral family (Microviridae) and one RNA viral family (Retroviridae). Overall, sequences significantly similar to Podoviridae were the most abundant in the *P. harrisoni* and *A. downingi* viromes. Various morphological types of virus-like particles (VLPs) were confirmed in the healthy coral tissue by transmission electron microscopy, including large tailless VLPs and electron-dense core VLPs. Tailed bacteriophages were isolated from coral tissue using a plaque assay. Higher functional gene diversity was recorded in *A. downingi* than in *P. harrisoni*, and comparative metagenomics revealed that the Gulf viral assemblages are functionally distinct from Pacific Ocean coral viral communities.

## Introduction

The field of coral virology remains in its infancy (Correa et al., 2016). Coral viruses remain the least-studied constituent of the coral holobiont, despite the fact that they are expected to infect and modulate all other members of the holobiont (for a review, see Vega Thurber and Correa, 2011). Viruses are able to influence living systems through both host mortality and horizontal gene transfer (Suttle, 2007) and are considered the largest reservoir of unexplored genetic diversity in the marine system (Monier et al., 2008). Presently, all available information about coral viruses is derived from studies performed on corals thriving in conditions of pristine warm water, where temperatures range between 16 and 32°C (Marhaver et al., 2008; Vega Thurber et al., 2008; Correa et al., 2013, 2016; Weynberg et al., 2014) and any increase or decrease in temperature outside the range causes coral to perish (Jokiel and Coles, 1990; Hoegh-Guldberg, 1999). No information is available about the viruses of corals that thrive naturally under extreme conditions, such as those dominating the Arabian Gulf.

The Arabian Gulf stands out among world seas because it is considered both one of the hottest seas and the most thermally varying coral-bearing water body in the world (Riegl and Purkis, 2012). The corals of the Arabian Gulf survive great fluctuations in water temperatures, which range from 39°C in summer to less than 11°C in winter (Spalding et al., 2001), making them a model system to study the impacts of rising global sea temperatures on corals (IPCC, 2007; Riegl and Purkis, 2012). In addition, as a semi-closed sea, the Gulf bears the direct impact of various human activities that increase the level of nutrients and pollutants in the system (Sheppard et al., 2010). It is well documented that coral reefs, which represent one of the world’s most productive marine ecosystems, are being rapidly depleted worldwide as a result of global and local disturbances that affect corals both directly and indirectly. The major global threats to coral reefs include climate change caused by global warming (Hoegh-Guldberg, 1999; Carpenter et al., 2008) and pollution (Hoegh-Guldberg, 2010). Recently, an important question has arisen regarding Arabian Gulf corals: how can Arabian Gulf corals survive fluctuations in water temperature better than corals elsewhere (see Riegl and Purkis, 2012)? Previous studies suggested that viruses might play a role in increasing the thermotolerance of their hosts similar to the case of Curvularia Thermal Tolerance Virus and its fungal host, *Curvularia protuberata* (Márquez et al., 2007). Also, Jacquet and Bratbak (2003) reported that phytoplankton *Micromonas pusilla* and *Phaeocystis pouchetii* became less sensitive to UVB when co-cultured with marine viruses. These findings support the idea that by transferring genetic material during viral infection, viruses can potentially enhance host metabolism, immunity, distribution, and evolution (Rohwer and Vega Thurber, 2009). Ongoing research to investigate the ability of corals to adapt to high temperature has focused either on the coral animal itself (Riegl and Purkis, 2012) or on the other members of the holobiont, especially *Symbiodinium* (Baker et al., 2004; Mostafavi et al., 2007; Shahhosseiny et al., 2011; Hume et al., 2013; Mahmoud and Al-Sarraf, 2016) and bacteria (Al-Dahash and Mahmoud, 2013; Mahmoud and Kalendar, 2016). Presently, no information is available about the viral assemblages of the Gulf corals.

The objective of this study was to determine the taxonomical and functional diversity of viruses in healthy Arabian Gulf corals. Therefore, we constructed and investigated two viral metagenomes to address major questions regarding the viral diversity associated with *Acropora downingi* and *Porites harrisoni*.

## Materials and Methods

### Sampling and Sample Sites

**FIGURE 1:**
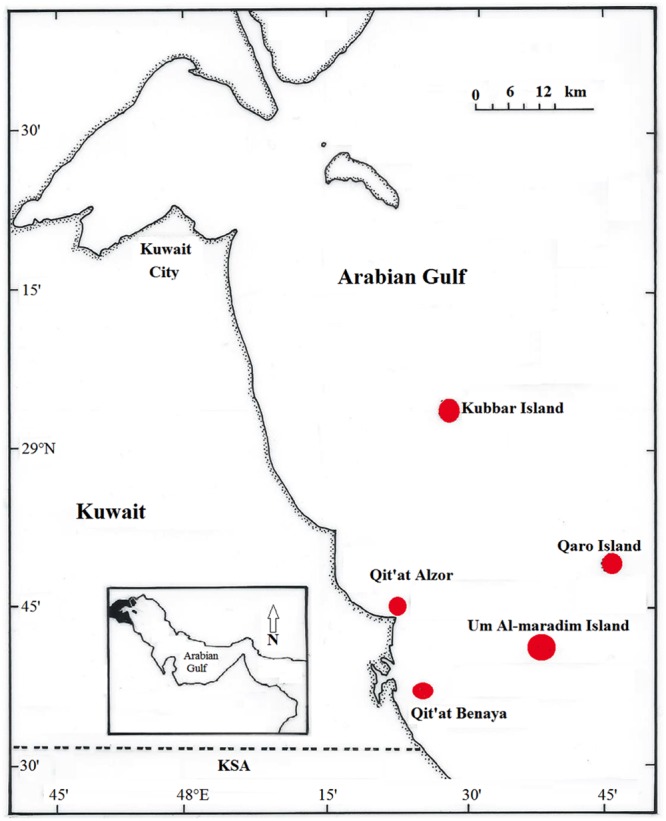
Kuwait map showing the three offshore reef systems (Kubbar Island, Qaro Island, and Umm Al Maradim Island) and the two inshore reef systems (Qit’at Alzor and Qit’at Benaya) sampled during the study.

Coral reefs in the Gulf are categorized into inshore and offshore reef systems. Inshore reefs are in close proximity to land and are greatly influenced by adjacent human activities, including sewage discharge, oil production and seawater desalination activities, whereas offshore reefs are less affected by such activities (Carpenter et al., 1997; Al-Ghadban et al., 2007). *A. downingi* and *P. harrisoni* nubbins were collected by SCUBA from two inshore locations, including the Qit’at Benaya [28.6164° N, 48.4289° E] and Qit’at Alzor reefs [28.7607° N, 48.3912° E], and three offshore locations, including the Umm Al Maradim Island [28.6851° N, 48.6568° E], Kubbar Island [29.0754° N, 48.4926° E] and Qaro Island [28.8161° N, 48.7747° E] reef systems (**Figure 1**). The sites are located in the Arabian Gulf directly south of Kuwait and were selected based on the availability of previous data regarding coral-associated microbes at these sites (Al-Dahash and Mahmoud, 2013; Mahmoud and Al-Sarraf, 2016; Mahmoud and Kalendar, 2016).

*Acropora downingi* and *P. harrisoni* were chosen because they are ubiquitous in the Arabian Gulf and are categorized in the International Union of Conservation of Nature and Natural Resources (IUCN) red list of threatened species either as being of least concern or not having been assessed. *A. downingi* is more fragile (i.e., more susceptible to thermal stress) than *P. harrisoni* and recovers from diseases at a slower rate (Guest et al., 2016). As a consequence, Acroporidae-dominated reefs often become Poritidae-dominated reefs after incidents of massive bleaching (Coles and Riegl, 2013).

From each site, three coral colonies of each type were selected, tagged and repeatedly sampled in July and October 2011 and in January 2012. The water quality for each site at each sampling occasion is included in Supplementary Table S1. The sampled coral colonies remained healthy throughout the entire study period and exhibited no signs of stress or bleaching. Coral nubbins were collected by cutting 1-cm pieces of the three tagged colonies of both *A. downingi* and *P. harrisoni* from each site using a sterile hammer and chisel for massive coral and cutters for branching coral. Using different sets of sampling tools for different coral types reduced cross-contamination between colonies, and divers were wearing gloves throughout the sampling process. In addition, three subsamples of each colony were collected on each sampling date. All samples were placed underwater in sterile sampling bags, and they were packed on ice and returned to the laboratory for immediate processing.

### Sample Processing

In the laboratory, the samples were divided into two sets. The first set was used for molecular analyses, whereas the second set was used for electron microscopy. The coral nubbins of the first set were washed with 0.02 μm-filtered sterile saline water (FSS, 3% NaCl) by placing coral nubbins in sterile stomacher bags and vigorously shaking them for 5 min. After shaking, samples were macerated in 10 ml of FSS using a sterile mortar and pestle (Vega Thurber et al., 2009b). The resulting slurry was liquefied with a syringe and used to extract viral DNA for metagenomic analysis. Coral nubbins of the second sample set were washed with FSS and immediately fixed in 2.5% v/v glutaraldehyde buffer (as described below).

### Transmission Electron Microscopy (TEM)

The coral nubbins were processed according to the method described in Patten et al. (2008). The coral nubbins were fixed with 2.5% v/v glutaraldehyde in 0.1 M sodium cacodylate buffer (pH 7.4). Then they were decalcified using a solution made of 10% EDTA in 0.03 M NaOH and dehydrated in 30 to 100% of acetone series diluted in water (Wilson et al., 2005). Samples were kept in the dark at 4°C throughout the decalcification and fixation process. The decalcified samples were post-fixed in 1% osmium tetroxide in cacodylate buffer before being embedded in Epon 812 epoxy resin (TAAB laboratories, United Kingdom), sectioned and imaged by TEM (JEOL JEM-1200EX II; JEOL, Japan). 70 nm thick sections were used and they were positively stained using Uranly acetate and Lead citrate. Micrographs were obtained using a digital Erlangshen ES1000W CCD camera (Gatan, United States) and Gatan Digital Micrograph software (United States).

### Isolation of Infectious Bacteriophages from Coral Using Plaque Assay (PA)

Bacteriophages associated with the coral nubbins in the current study were isolated using pure bacterial cultures. More than 100 different bacterial species were isolated from the two coral genera tissue and mucus from 2009 to 2012 (Al-Dahash and Mahmoud, 2013; Mahmoud and Kalendar, 2016). Coral nubbin homogenized slurry was filtered through sterile 0.22-μm Nalgene sterile bottom-top filter units (Sigma–Aldrich, United States), followed by a series of dilutions to bring the phage numbers to 20–200 viruses per aliquot as determined using SYBR green and epifluorescence microscopy (Noble and Fuhrman, 1998). The plaque assay was conducted following the protocol of Wilhelm and Poorvin (2001) (Supplementary Data), and bacteriophages were visualized using TEM following the protocol of Ackermann (2009).

### Virus Purification and Concentration

To purify and concentrate viruses from coral samples, the liquefied coral-nubbin slurries were homogenized in a stomacher bag for 2 min. The resulting mixtures were filtered through 47 mm-diameter 8 μm-pore size nucleopore polycarbonate Whatman filters (Fisher Scientific, United States). The filtrate was centrifuged at 1000 × *g* for 15 min to remove the host cells and the coral skeleton. The supernatant was then sequentially filtered through 0.45- and 0.22-μm Nalgene sterile bottom-top filter units (Sigma–Aldrich, United States). The presence of viruses in the filtrate was confirmed using SYBR green and epifluorescence microscopy (Noble and Fuhrman, 1998). Viruses in the filtrate were concentrated using CsCl gradients according to the protocol described in Vega Thurber et al. (2009a). Briefly, CsCl concentrations of 1.7, 1.5, 1.35, and 1.15 g ml^-1^ were added to clear ultracentrifuge tubes. Next, identical volumes of the samples were added to the top of the created gradient. The tubes were centrifuged at 60,000 × *g* for 2 h at 4°C in a swinging-bucket rotor using a refrigerated Optima L-100 XP ultracentrifuge (Beckman Coulter, United States). The virions were collected using a sterile 18-gauge needle. The mouth of the needle was placed immediately below the 1.5 g ml^-1^ step, and viruses from the 1.3 to 1.5 g ml^-1^ fraction were collected. Samples were assessed with SYBR green and epifluorescence microscopy to confirm the presence of virions.

### Viral DNA Extraction, Amplification and Sequencing

To remove naked DNA from each sample, 2.5 U of DNase I (Sigma–Aldrich, United States) was added per 100 μl of sample, and the mixture was incubated for 1 h at 37°C (Vega Thurber et al., 2009a). Both DNase I and RNA were heat-denatured by incubating the mixture at 70°C for 10 min. The viral DNA was then extracted using the QIAamp MinElute Virus Spin Kit (Qiagen, United States) by following the manufacturer’s protocol. The DNA concentration was determined, and DNA purity was confirmed using a Nanodrop nucleic acid analyser (Thermo Scientific, United States). The DNA of all the samples of a given coral host was pooled; this step facilitates the examination of a broader diversity of coral-associated viruses, as suggested by Marhaver et al. (2008).

The removal of bacterial and eukaryotic DNA from the extracted viral DNA was confirmed by screening the viral DNA samples for absence of amplification products with universal primers for 16S and 18S rDNA genes (Schafer and Muyzer, 2001).

A random-amplification PCR protocol described by Wang et al. (2003) was adapted for this work. A mixture containing 7 μl of genomic DNA (450 ng), 2 μl 5X Sequenase Buffer (13 units μl^-1^) (United States Biochemical, United States), and 1 μl Primer A (40 pmol μl^-1^) [GTT TCC CAG TCA CGA TCN NNN NNN NN] was incubated in a Gene Amp PCR system 9700 thermocycler (Applied Biosystems, United States) at 94°C for 2 min before being rapidly cooled to 10°C and held at 10°C for 5 min. Next, a sequenase mix (2.0 μl 5X Sequenase Buffer, 7.7 μl H_2_O, and 0.3 μl Sequenase) was prepared and added to the PCR tubes to give a total volume of 30 μl. The thermocycler temperature was ramped from 1 to 37°C over 8 min, at which point the temperature was maintained at 37°C for an additional 8 min. The temperature was then rapidly increased to 94°C and held for 2 min. The temperature was then decreased to 10°C and held for 5 min while 1.2 μl of diluted Sequenase (1:4 dilution) was added. The temperature was then increased from 10 to 37°C over 8 min and held for an additional 8 min before being increased to 94°C for an additional 8 min (Wang et al., 2003).

The resulting samples were amplified in 0.2-ml PCR tubes containing 6 μl of the produced DNA template, 4 μl of 50 mM MgCl_2_, 10 μl of 10X PCR Buffer, 1 μl of 25 mM dNTPs, 100 pmol μl^-1^ Primer B [GTTTCCCAGTCACGATC], 1 μl Taq polymerase (5 units μl^-1^) (hot start) and 77 μl water. The PCR program included 35 cycles of 30 s at 94°C, 30 s at 40°C, 30 s at 50°C and 1 min at 72°C. PCR amplicons were cleaned using a PCR clean-up spin kit (Qiagen, United States).

The samples were sequenced by the Yale Center for Genome Analysis facility (Connecticut, United States). Prior to sequencing, the samples were assessed for integrity on a bioanalyser and fragmented to ∼500 nt. The samples were sequenced on an Illumina HiSeq 2000 instrument using 76-nt paired-end sequencing.

### Bioinformatics and Statistical Analysis

Prior to their assembly and annotation, reads were trimmed to remove primers using TagCleaner version 0.12 (Schmieder et al., 2010) and quality-trimmed for a minimum phred score of 20 using Trimmomatic version 0.36 (Bogler et al., 2014). The non-viral (non-target) sequence reads were identified and removed prior to assembly of contigs (Supplementary Data). All putative viral sequences were then assembled using Velvet version 1.2.10 (Zerbino and Birney, 2008) with *k*-mer lengths of 39 and 49. A final hybrid assembly was generated using Minimus II version 3.1.2 (Sommer et al., 2007) from Amos version 3.10 with a minimum overlap of 20 bp and 98% identity, and genes were predicted by FragGeneScan (Min et al., 2010). Viromes were annotated with a tBLASTx and BLASTp search (threshold *E*-value 10^-5^) against the GenBank viral non-redundant (nr and pep) and NCBI viral refseq databases (and protein database) and with MG-RAST version 3.2.45 (Meyer et al., 2008).

Variations in functional diversity between the Gulf coral viromes and Pacific coral viromes (Vega Thurber et al., 2009b) were determined using MG-RAST principal coordinates analysis (PCoA) and using XIPE-TOTEC software (Rodriguez-Brito et al., 2006). To produce MG-RAST PCoA for the compared viromes, the data were first normalized using DEseq (Anders and Huber, 2010) and then analyzed using Bray–Curtis distances using tools available through MG-RAST. Within XIPE-TOTEC, a non-parametric analysis of variance (ANOVA) was used to determine which metabolic subsystems in the MG-RAST data were significantly over- or underrepresented in the compared viromes. The Shannon index of diversity and sequence evenness scores for functional genes were calculated by following the protocol described in Dinsdale et al. (2008). The Gulf coral viral metagenome projects were submitted to GenBank under the following accession numbers: *P. harrisoni* (SRR5452095) and *A. downingi* (SRR5452096). Also the two viromes are submitted to MG-RAST under the following accession numbers: *P. harrisoni* (mgm4767658.3) and *A. downingi* (mgm4767672.3).

## Results

### Taxonomic Composition of the Two Gulf Coral Viromes

In total, 110,866,698 bp and 141,093,140 bp of raw read data were generated from *A. downingi* and *Porites harrisoni*, respectively, and an average of 21% of the reads were quality trimmed. The number of reads that remained from *A. downingi* and *P. harrisoni* after trimming was 81,703,324 and 118,925,078 bp, respectively. There was very little contamination with cellular gene markers, as demonstrated by the low number of matches to ribosomal proteins (0.3%). On average, 73% of the sequences in the two libraries did not match any nucleotide sequences in the NCBI database, based on tBLASTx analysis. Among the 17% of sequences in the two viromes that matched nucleotide sequences in the NCBI database, the majority of these sequences (97%) exhibited similarity to prokaryotic and eukaryotic genes rather than to viral genes, and only 2.3% were classified as viruses. The same results were confirmed using MG-RAST software.

**FIGURE 2:**
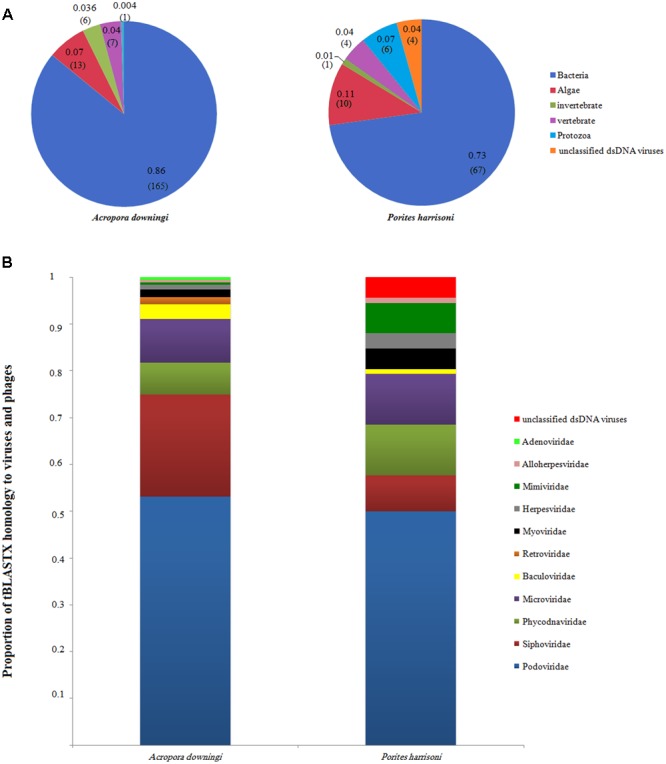
Annotated sequence classification according to tBLASTx hits in the GenBank non-redundant database (*E*-value 10^-5^). **(A)** Groups, percentage and proportion of viral annotated sequences after excluding eukaryote and prokaryote genes. The number of contigs for each group is also included between brackets. **(B)** The proportion of tBLASTX homology to virus and phage families in *Acropora downingi* and *Porites harrisoni* viromes.

The total number of viral reads from *A. downingi* that remained after removing the non-viral sequence reads was equal to 1,768,711 bp, which corresponded to 380 contiguous sequences with an average length of 204 bp and a maximum length of 3,257 bp; in *P. harrisoni*, the virome library was composed of 2,970,666 bp, with 321 contigs of an average length of 174 bp and a maximum length of 3,209 bp. The taxonomic compositions of the viral assemblages in the two viral metagenomes were determined (**Figure 2**). The tBLASTx results (**Figures 2A,B**) revealed that the two coral viromes contained 12 viral families, including ten dsDNA viral families [Caudovirales (Siphoviridae, Podoviridae, Myoviridae), Phycodnaviridae, Baculoviridae, Herpesviridae, Adenoviridae, Alloherpesviridae, Mimiviridae and one unclassified family], one ssDNA viral family (Microviridae) and one RNA viral family (Retroviridae).

The *A. downingi* viral assemblages were mainly dominated by viruses that target phages (86%, 165 contigs). Algal viruses (7%, 13 contigs) were the second most dominant group in this virome, followed by vertebrate (4%, 7 contigs), invertebrate (3%, 6 contigs) and protozoa (0.3%, 1 contig) viruses. On the other hand, phages (73%, 67 contigs) and algae (11%, 10 contigs), protozoa (7%, 6 contigs), vertebrate and unclassified (4%, 4 contigs), and invertebrate viruses (1%, 1 contig) dominated the *P. harrisoni* virome (**Figure 2A**). **Figure 2B** shows the variation within the detected viral families in *A. downingi* and *P. harrisoni*: Podoviridae (53.1%, 102 contigs), Siphoviridae (21.8%, 42 contigs), Microviridae (9.4%, 18 contigs) and Phycodnaviridae (6.8%, 13 contigs) dominated the *A. downingi* virome, while Podoviridae (52.3%, 46 contigs), Phycodnaviridae and Microviridae (11.4%, 10 contigs each) and Siphoviridae (6.8%, 7 contigs) dominated the *P. harrisoni* virome.

### Metabolic Profiles of Gulf Coral Viromes and Comparison with Other Coral Viromes

**FIGURE 3:**
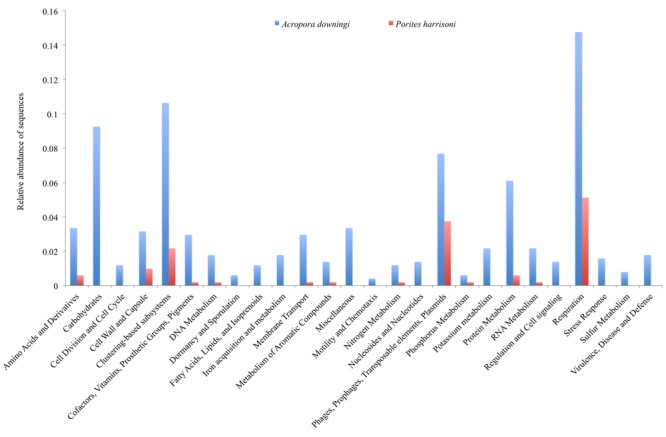
Relative functional gene sequences abundance in Arabian Gulf *A. downingi* and *P. harrisoni* viromes. Protein-encoding sequences were analyzed using BLASTX (*E*-value cut-off-10^-5^) to the MG-RAST subsystem database and grouped according to the highest hierarchal metabolic function or ‘subsystem.’

The metabolic profiles of the two Gulf coral viromes were explored using MG-RAST, which assigns sequences to metabolic categories based on the best BLASTx hit against the subsystem database (*E*-value < 10^-5^). The most-represented categories in the *A. downingi* virome were related to respiration, then cluster-based subsystems, carbohydrate and phages, in that order. In *P. harrisoni*, the most-represented categories, in descending order, were respiration, phages and cluster-based subsystems (**Figure 3**).

Thirteen categories, including carbohydrate, stress responses, virulence, disease and defense, regulation and cell signaling, motility and chemotaxis, cell division and cell cycles, dormancy and sporulation, and metabolisms (fatty acids, lipids, isoprenoids, iron, potassium, and nucleosides and nucleotides) were detected only in *A. downingi* and not in *P. harrisoni* (**Figure 3**). The carbohydrate category had a higher total abundance of sequences than the rest of the categories. Based on the functional genes obtained from MG-RAST, the virome of *A. downingi* (2.88) exhibited greater functional diversity (H′) than that of *P. harrisoni* (2.77), whereas the functional evenness of both coral viromes was 0.11.

**FIGURE 4:**
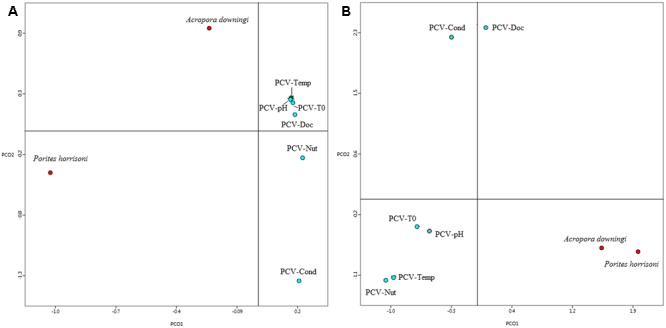
First two principle coordinates from the principal coordinate analysis of the viral communities from the Gulf coral viromes and Pacific coral virome (Vega Thurber et al., 2009b) as produced from the MG-RAST analysis for the **(A)** functional genes where protein-encoding sequences were analyzed using BLASTX (*E*-value < 10^-5^) to the subsystem database and grouped according to the highest hierarchal metabolic function or ‘subsystem. PCV1, pacific coral virome generated from stressed coral holobiont project; Temp, temperature; T0, control; Nut, nutrient; Cond, conductivity; DOC, dissolved organic carbon. **(B)** Best hit of viral classification.

A comparison in MG-RAST of the two Gulf coral viromes with 6 coral viromes that represent Pacific Ocean corals (Vega Thurber et al., 2009b) revealed clear segregation between the Gulf coral viromes and the other viromes (**Figure 4**). The coral viromes were compared based on the metabolic subsystems in MG-RAST; the resulting PCoA exhibited clear clustering of the Gulf coral viromes together and a clear distinction from the virome of *P. compressa* that inhabits the Pacific Ocean (Vega Thurber et al., 2009b) (**Figure 4**). A comparison of the *P. harrisoni* and the *A. downingi* viromes with the Pacific Ocean coral viromes using XIPE-TOTEC revealed significant overrepresentation of phage and prophage sequences in the Gulf coral viromes (*P* < 0.002) relative to those in the Pacific Ocean coral viromes. This comparison is clear in Supplementary Figure S1. **Table 1** summarizes some of the sequences in the Gulf coral viromes with similarities to phage and prophages.

**Table 1:** Some of the gene annotations from the Arabian Gulf *Porites harrisoni* and *Acropora downingi* viromes showing those with similarities to phage and prophage sequences.

Viral family (viridae)	Host	Accession #	Contig coverage	nt size	aa% Identity	*E*-value	Virus genome similarity	Gene annotation
Podo	*Acropora*	NC_001422.1	29038	3257	99	0	*Enterobacteria* phage phiX174 sensu lato	DNA replication initiation protein gpA
Micro	*Acropora*	NC_004821.1	239	1494	88	0	*Bacillus* prophage phBC6A52	Hypothetical protein BC2597
Micro	*Acropora*	NC_007817.1	12334	729	63	5e-96	*Enterobacteria* phage ID2 Moscow/ID/2001	Internal scaffolding protein
Sipho	*Acropora*	NC_008371.1	1131	511	93	1e-95	*Lactococcus* phage jj50	Hypothetical protein LPJV50_ORF31
Sipho	*Acropora*	NC_017688.1	768	497	80	7e-60	*Lactococcus* Phage ASCC191	Hypothetical protein LLAPH_191_0037
Sipho	*Acropora*	NC_001416.1	76	446	100	1e-91	*Enterobacteria* phage lambda	Putative single-stranded DNA binding protein
Podo	*Porites*	NC_001422.1	41317	3209	100	0	*Enterobacteria* phage phiX174 sensu lato	Minor spike protein
Micro	*Porites*	NC_004821.1	1472	1041	98	0	*Bacillus* prophage phBC6A52	Portal protein
Myo	*Porites*	YP_004323946.1	49	226	97	2e-39	*Synechococcus* phage Syn19	Hypothetical protein Syn19_113
Sipho	*Porites*	YP_009277994.1	31	229	99	1e-44	*Propionibacterium* phage QueenBey	Putative minor tail protein
Sipho	*Porites*	YP_009152377.1	25.72	230	99	7e-47	*Propionibacterium* phage PHL092M00	Putative protease

*Annotations are based on tBLASTx to the NCBI viral database (*e*-value threshold 10^-*5*^).*

### Electron Microscopic Analysis of Gulf Coral Tissue

**FIGURE 5:**
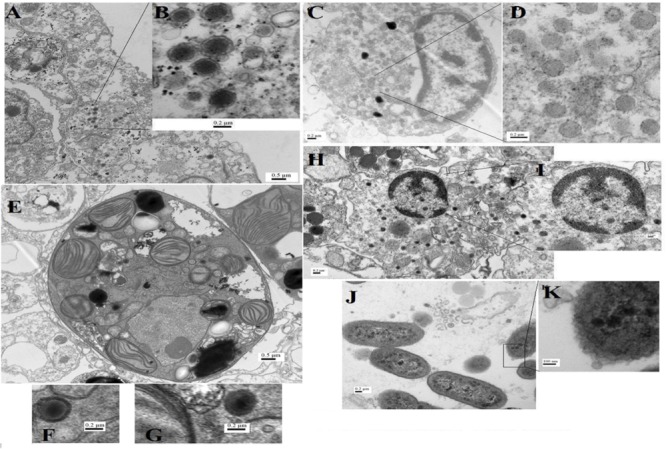
Transmission electron microscopy (TEM) images for virus-like particles **(A–H)** with sizes ranging from 75 to 200 nm and phages **(J,K)** from *A. downingi* and *P. harrisoni* tissue. **(A,B)** Large tailless nucleocytoplasmic VLP particles from epithelial cells of *A. downingi*. **(C,D)** Virus factory from epithelial cells. **(E–G)** *Symbiodinium* cell within the gastrodermis layer of *A. downingi* with large VLP (>300 nm). **(H,I)** VLP rupturing host nucleus and release of Herpes-like viral particles from *P. harrisoni* samples. **(J,K)** Tailless VLP attached to prokaryote cell within *A. downingi* tissue.

**FIGURE 6:**
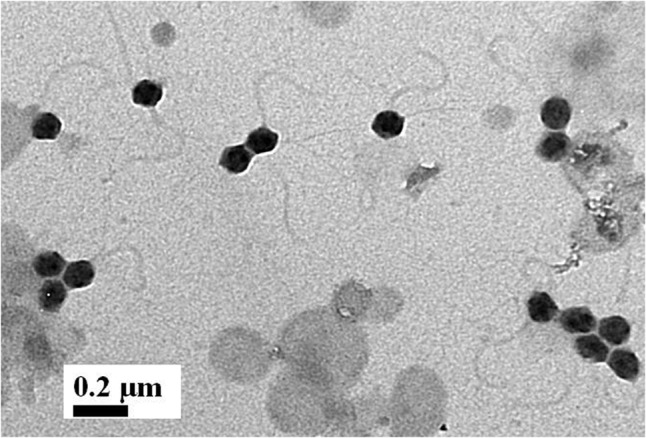
Electron micrographs showing vibriophages isolated from *A. downingi* using plaque assay, positively stained with 2% uranyl acetate solution and photographed at X60000 magnification.

Despite being the most dominant predicted viral group in the *P. harrisoni* and *A. downingi* virome, the presence of tailed bacteriophages was not detected inside coral tissue using electron microscopy. In contrast, most of the viruses that were detected in the coral tissue (**Figure 5**) and were identified as infecting prokaryotic and eukaryotic members of the holobionts were tailless viruses with or without envelope and with average sizes ranging from 75 to 300 nm. Various morphological types of virus-like particles (VLPs) were found in the healthy coral tissue by TEM and included large tailless VLPs and electron-dense core VLPs (**Figure 5**). The current work documents the presence of giant VLPs (>200 nm) in the tissue of *P. harrisoni* and *A. downingi* (**Figures 5A–G**). **Figures 5C,D** show what is known as the “virus factory,” which characterizes giant viruses known as the nucleocytoplasmic large viruses (NCLDV). In addition, giant VLPs within *Symbiodinium* cells are represented in **Figures 5E–G**, where the VLP size reached >300 nm. Electron-dense core VLPs with sizes ranging from 150 to 210 nm and with enveloped, icosahedral (non-tailed) capsids may be Herpes-like viral particles observed in the cell nuclei of both corals (**Figures 5H,I**). In contrast, tailed bacteriophages (Sipho-, Podo-, and Myo-) were not detected in the examined coral tissue by TEM. However, TEM images from *A. downingi* suggest that a tailless VLP with an icosahedral capsid 75 nm in diameter is infecting rod-shaped bacteria that inhabit the coral tissue; there is a possibility that this VLP could be a Podoviridae with a tail adsorbed to the host surface (**Figures 5J,K**). Tailed vibriophages were isolated from both *A. downingi* and *P. harrisoni* coral tissue using a phage plaque assay and cultures of a *Vibrio* sp. that were previously obtained from tissue of the same coral types (Al-Dahash and Mahmoud, 2013; Mahmoud and Kalendar, 2016). The tailed bacteriophage that were isolated from *A. downingi* tissue showed morphological features similar to members of the Siphoviridae family that have an isometric head and a long thin, flexible and non-contractile tail (**Figure 6**).

## Discussion

Few studies have focused on coral viromes (Marhaver et al., 2008; Vega Thurber et al., 2008, 2009b; Correa et al., 2013; Weynberg et al., 2014; Wood-Charlson et al., 2015; Correa et al., 2016), and they all concern corals from temperate and tropical regions that thrive in temperatures ranging from 16 to 32°C. We provide here the first analysis of viral communities in corals that naturally thrive in a thermally variable water body, where temperatures fluctuate between 11 and 39°C in different seasons.

Despite the stringent preparation protocols for the Gulf coral viromes and the low number of reads that were annotated as ribosomal RNA (an indicator of cellular contamination) in the prepared viromes, the two Gulf coral viromes were dominated by unknown sequences. As in studies of viromes that were obtained from different environments (Zhang et al., 2006; Fierer et al., 2007; Schoenfeld et al., 2008; Rosario et al., 2009; Parsley et al., 2010; Bibby et al., 2011; Park et al., 2011), including corals, the majority of the sequences had no homology to known genes of either viral or cellular origin and were classified as unknown (Mokili et al., 2012). This finding may suggest an insufficient representation of phages and viruses in the databases, implying that the majority of the viruses in these habitats remain uncharacterized (Culley, 2011). The majority of the known sequences (97%) were annotated to cellular organisms, including both prokaryotes and eukaryotes, due to host-derived genes, thus reflecting the “genetic mosaicism” of viruses (Vega Thurber et al., 2008, 2009b; Marhaver et al., 2008; Fancello et al., 2012).

The viral groups detected in Gulf corals were previously reported in corals elsewhere (Wood-Charlson et al., 2015). We caution that the taxonomic and functional analysis of viromes in this study is based on a small number of contigs. Keeping this in mind we find that the variation in the viral assemblages of the two most important coral types inhabiting the north Arabian Gulf (i.e., *A. downingi* and *P. harrisoni*) is evident in our study. The number of viral families that were identified in the *A. downingi* virome was greater than that found in *P. harrisoni*. The identified *A. downingi* and *P. harrisoni* viral sequences were dominated by sequences from phages, with the second most abundant sequences being from algal viruses, and the most-represented viral family in both corals was Podoviridae. This finding opposes the report of *A. aspera* that inhabit Heron Island, whose most dominant phage was Siphoviridae and least dominant was Podoviridae (Correa et al., 2016). However, we have to take into consideration that the Gulf viromes were produced by processing the entire coral nubbin, while in Correa et al. (2016), the viromes were produced by blasting only the coral tissue from the skeleton using an airbrush, which was followed by processing the blastate. This means that the Gulf coral viromes may include viruses that target other coral endolithic hosts. A previous study in *P. astreoide*s that used full coral nubbins reported that the number of sequences with significant similarities to Podoviridae was the least abundant among dsDNA phage families in the produced metagenome (Wegley et al., 2007). On the other hand, Vega Thurber et al. (2008) showed that the Pacific Ocean *P. compressa* virome that was produced from whole coral nubbins was dominated by phages and that >60% of the viral sequences that were identified as eukaryotic viruses were similar to vertebrate viruses instead of algal viruses. Can the dominance of Podoviridae, which are phage with lytic lifestyles, in Gulf corals be related to coral strategies for surviving harsh conditions by controlling their symbiont density (Cunning and Baker, 2012)?

Only 12.7% of the identified viral sequences in the two viromes matched large viral families (i.e., Phycodnaviridae and Mimiviridae), despite the fact that the TEM images for the two coral tissues suggested that they are more abundant. This result may reflect the magnitude of discrimination against the genomes of these important viruses due to the method that was used. The viral isolation methods used in the present study succeeded in removing genomic contamination from cellular members of the holobionts, but the filtration step may have discriminated against large viruses. The importance of NCLVs to corals cannot be neglected. Martínez et al. (2014) stated that giant viruses are neither rare nor marginal players in marine ecosystems, whereas Correa et al. (2013) provided genomic evidence for the dominance of nucleocytoplasmic large DNA viruses (Mimiviridae and Phycodnaviridae) in *Montastraea cavernosa* and its algal endosymbiont. In addition, Correa et al. (2016) emphasized the importance of jointly applying electron microscopy and metagenomics when identifying dominant or core viral types, particularly NCLVs, within coral samples. In the current study, the TEM images of both *A. downingi* and *P. harrisoni* indicated the presence of giant VLPs (≥200 nm) in different parts of the healthy coral tissue and inside *Symbiodinium* cells (**Figures 5E–G**), raising questions about their role and importance in healthy Gulf corals. Our TEM and metagenomic data suggest the presence of giant VLPs, some of which are characterized by a Mimiviridae-like shape in the Gulf coral tissue (personal communication with Hans Ackermann; Correa et al., 2016). Claverie et al. (2009) reported that Mimiviridae are the second most abundant group in the marine system, exceeded only by bacteriophages. They suggested that ancestral Mimiviridae play a role in constructing octocorals’ mitochondrial genomes, as Mimiviridae exist in close proximity to corals and are able to infect coral tissue.

Our study also provides documentation for what is known as the virus factory (**Figures 5C,D**) within Gulf coral epidermal cells. These factories are created *de novo* in the viral host upon infection. Virus factories and large VLP particles that were in the cytoplasm of coral epithelial cells and associated with the *Symbiodinium* in the gastrodermal region were detected by TEM in the tested healthy Gulf corals. The images that we obtained (**Figure 5**) are similar to others that were previously published by Correa et al. (2016). Therefore, the role of NCLV in healthy Gulf coral tissue warrants further investigation.

Herpesviridae family sequences were found in the viromes of both Gulf corals. Furthermore, Herpes-like particles were observed in the cell nuclei of Gulf corals using TEM (**Figures 5H,I**). According to Vega Thurber et al. (2008), stressors induce production of Herpes-like viruses in Pacific Ocean corals; they found that the quantities of Herpes-like viral sequences increased up to six orders of magnitude in *P. compressa* that were exposed to high temperatures in comparison to their control counterparts. In addition, Correa et al. (2016) stated that Herpes-like annotations dominate dsDNA viral taxa in corals and that the most commonly identified VLPs in TEM images of both *A. aspera* and *A. millepora* tissue were particles with morphology highly reminiscent of herpesviruses. The viromes and the TEM images of Gulf corals do not support the previous findings.

Sequences significantly similar to Retroviridae members were found only in the *A. downingi* viral sequences. Retroviruses and RNA viruses with intermediate DNA stages are typically detected in DNA viral metagenome libraries (Correa et al., 2013, 2016; Weynberg et al., 2014; Wood-Charlson et al., 2015). Garcia et al. (2013) previously reported an abundance of retroviruses in the microbiomes of healthy and diseased *Mussismilia braziliensis* and *A. millepora* from the Atlantic Ocean. Additionally, Correa et al. (2013) revealed a high abundance of retroviruses in healthy and stressed *Montastraea cavernosa*, a common Caribbean reef-building coral. Recently, Correa et al. (2016) showed that retroviruses were the most dominant type of eukaryotic virus in the virome of *A. aspera* and that VLPs with physical shape similarities to retroviruses were detected by TEM in *A. aspera* tissue. Rohwer and Vega Thurber (2009) stated that retroviruses are potential vectors for horizontal gene transfer between animals and plants; in our case, such transfer might occur between coral cells and their algal symbiont. The presence of retroviruses in the *A. downingi* virome compared with the lack of retroviruses that were found in *P. harrisoni* in the current study may reflect physiological differences between the two corals (Gates and Ainsworth, 2011). However, the question of the role of retroviruses in corals remains unanswered.

The important roles of viruses as genomic manipulators of both prokaryotic and eukaryotic members of the coral holobiont are revealed by our deeper investigations into the functional diversity of annotated proteins within the Gulf coral viromes. *A. downingi* exhibited somewhat greater diversity in its functional genes than those of *P. harrisoni* did, and the two investigated viromes exhibited low evenness, reflecting the dominance of relatively few metabolic pathways in Gulf corals. Dinsdale et al. (2008) reported that coral-associated microbes generally exhibit low functional diversity because microbes associated with corals are taxonomically diverse.

Phage- and prophage-related genes were the second most dominant in *P. harrisoni* and the fourth most dominant in *A. downingi*. According to Bettarel et al. (2013), prophages protect their hosts from infection by a large number of related phages; this effect is considered vital for beneficial bacterial symbionts in coral holobionts. On the other hand, clustering-based subsystem-related genes were the second most dominant in the *A. downingi* and were the third most dominant in *P. harrisoni*. This finding may suggest the presence of functional coupling between certain genes, but without the knowledge of their precise functions, this point requires further investigation. The high occurrence of respiration-associated genes in the Gulf coral viromes may reflect the role of viruses in transferring this important group of genes that is responsible for host survival in the coral holobiont in environments in which the oxygenation diurnally fluctuates (Dinsdale et al., 2008) and, during summer, the increasing temperature decreases the oxygen solubility (Matear et al., 2000).

Finally, it was essential in the current study to compare the Gulf coral viromes with corresponding samples from corals inhabiting other, non-thermally fluctuating water bodies. When the two Gulf coral viromes were compared with six *P. compressa* viromes from the Pacific Ocean, they clustered separately based on functional gene and taxonomical diversity data obtained from MG-RAST. This finding supports the previous theory that viral assemblages differ in different coral species and in different geographical regions due to selective local pressures. However, we have to take into consideration the possibility that the separation noticed between the Gulf and Pacific coral viromes may be due to the differences in the algorithms applied by MG-RAST to analyze the compared viromes at the time of submission. In addition, Gulf coral viromes were produced by pooling samples collected from different seasons and sites together, which will add to the overall variability of these samples in comparison to the Pacific Ocean coral viromes, which were collected during Spring from one location and then exposed to different stressors under laboratory conditions. The XIPE-TOTEC results showed that Gulf corals exhibited over-representation of phage and prophage genes compared with the Pacific Ocean *P. compressa* virome, reflecting the importance of bacteriophages in the Gulf coral holobiont and their roles as top-down controllers of the diversity of coral-associated microbes (Wegley et al., 2007; Marhaver et al., 2008; Littman et al., 2011).

## Conclusion

Our results confirm that healthy corals that thrive naturally in thermally fluctuating water bodies contain diverse groups of phages and viruses, including giant viruses, and that the presence of viruses is not necessarily related to the onset of infection and disease in corals. Further studies are needed to reveal the roles of viruses in maintaining the holobiont balance in the Gulf corals so that hypotheses can be formulated about how Gulf corals survive harsh environmental conditions.

## Author Contributions

HM: initiator of the work, participate in all the practical involved in the work and run the metagenome practical fully, analyzed the metagenomes, prepared and wrote the manuscript. LJ: electron microscopic and phage work.

## Conflict of Interest Statement

The authors declare that the research was conducted in the absence of any commercial or financial relationships that could be construed as a potential conflict of interest.
