# Supplementary material for: Phage and Nucleocytoplasmic Large Viral Sequences Dominate Coral Viromes from the Arabian Gulf
Source: Front Microbiol. 2017 Oct 24;8:2063. doi: 10.3389/fmicb.2017.02063 (PMC5660727; doi:10.3389/fmicb.2017.02063)
Supplement: Supplementary file 1 [file Table_1.doc]

| Sampling dates  - Sites | Seawater quality parameters | | | |
| --- | --- | --- | --- | --- |
|  | pH | Conductivity  ms cm ^-1^ | Temperature  (°C) | Salinity  (%) |
| July 2011   - Qit’at Benaya - Qit’at Alzor - Um Al-maradim Island - Kubbar Island - Qaro Island | 8.47  8.44  8.38  8.29  8.42 | 60.5  60.9  60  60.7  58.2 | 33.8  31.8  31  31.4  31.5 | 4  4  4  4  3.94 |
| October 2011   - Qit’at Benaya - Qit’at Alzor - Um Al-maradim Island - Kubbar Island - Qaro Island | 8.45  8.39  8.45  8.47  8.47 | 59.2  59.6  58.6  60.1  59.4 | 29.2  28.8  28.1  29.2  30 | 3.96  3.99  3.91  4  3.96 |
| January 2012   - Qit’at Benaya - Qit’at Alzor - Um Al-maradim Island - Kubbar Island - Qaro Island | 8.34  8.23  8.31  8.48  8.87 | 58.58  59.29  58.27  60.5  59.7 | 16  13  18  16.5  17 | 4.02  4.14  4.2  4  3.93 |

**Supplementary data:**

The seawater quality variables at the day of sampling from all inshore and offshore reef systems are summarized in Table S1.

Table S1. Water quality variables from inshore and offshore reef systems at the time of sampling. Samples were collected in July and October 2011, and January 2012
