# Supplementary material for: Phage and Nucleocytoplasmic Large Viral Sequences Dominate Coral Viromes from the Arabian Gulf
Source: Front Microbiol. 2017 Oct 24;8:2063. doi: 10.3389/fmicb.2017.02063 (PMC5660727; doi:10.3389/fmicb.2017.02063)
Supplement: Supplementary file 2 [file Presentation_1.PDF]

Supplementary 3

A. Plaque assay protocol (bacteriophage Isolation)

Bacteriophages from coral nubbins were isolated using a selective-enrichment technique. The coral nubbin homogenized slurry was filtered through sterile 0.22-µm Nalgene sterile bottom-top filter units (USA). The filtrate was incubated with various strains of bacteria. The lytic activity of the enrichment supernatant was tested in soft Lauria-Bertani agar (Sigma, USA) inoculated with each of the bacteria strains under test by spot test verification.

In order to obtain pure phage isolates from the enriched supernatant, standard serial dilution purification procedure was done where:

1) Phages from enriched supernatant were diluted 10^{-2} to 10^{-4} in phage buffer.

2 0.5 ml pure bacterial broths were infected with 10 µl of each diluent and incubated at room temperature for 30 minutes.

3) 4.5 ml top agar was added to the mixture and poured on agar plates.

5) The plates were incubated at 30°C overnight.

Different size plaques were picked and purified by following the serial steps for three more times until a pure phage lysate was obtained.

B. The method applied to remove the non-target viral sequences:

-The sequences were published by the NCBI on the 18/4/2017.

- Complete reference viral genomes from NCBI were used. Recently, this reference contains 7144 complete viral genomes. All *Acropora downingi and Porites harrisoni* sequences were mapped against this reference using BWA version 0.7.12-r1039 (http://bio-bwa.sourceforge.net/). Default settings were used for the mapping process *(minimum seed length=19; band width for banded alignment=100, off-diagonal X-dropoff=100, look for internal seeds inside a seed longer than {-k} * FLOAT =1.5, seed*

*occurrence for the 3rd round seeding=20, skip seeds with more than INT occurrences=500, drop chains shorter than FLOAT fraction of the longest overlapping chain=0.50, discard a chain if seeded bases shorter than INT=0, perform at most INT rounds of mate rescues for each read=50, penalty for a mismatch=4, gap open penalties for deletions and insertions=6,6, gap extension penalty; a gap of size k cost '{-O} + {-E}*k =1,1, penalty for 5'- and 3'-end clipping =5,5, penalty for an unpaired read pair=17,...)*. Subsequently, the *de novo* assembly was proceeded with only those reads, that had aligned with the reference viromes. Reads that mapped to the references was extracted using samtools version 1.3.1 (http://samtools.sourceforge.net/)
