# Supplementary material for: Phage and Nucleocytoplasmic Large Viral Sequences Dominate Coral Viromes from the Arabian Gulf
Source: Front Microbiol. 2017 Oct 24;8:2063. doi: 10.3389/fmicb.2017.02063 (PMC5660727; doi:10.3389/fmicb.2017.02063)

Supplementary data

Figure S1. The relative abundance of sequences with similarities to phage and prophages in Gulf coral viromes and *Porites compressa* virome from the Pacific Ocean based on functional gene analysis using MG-RAST.

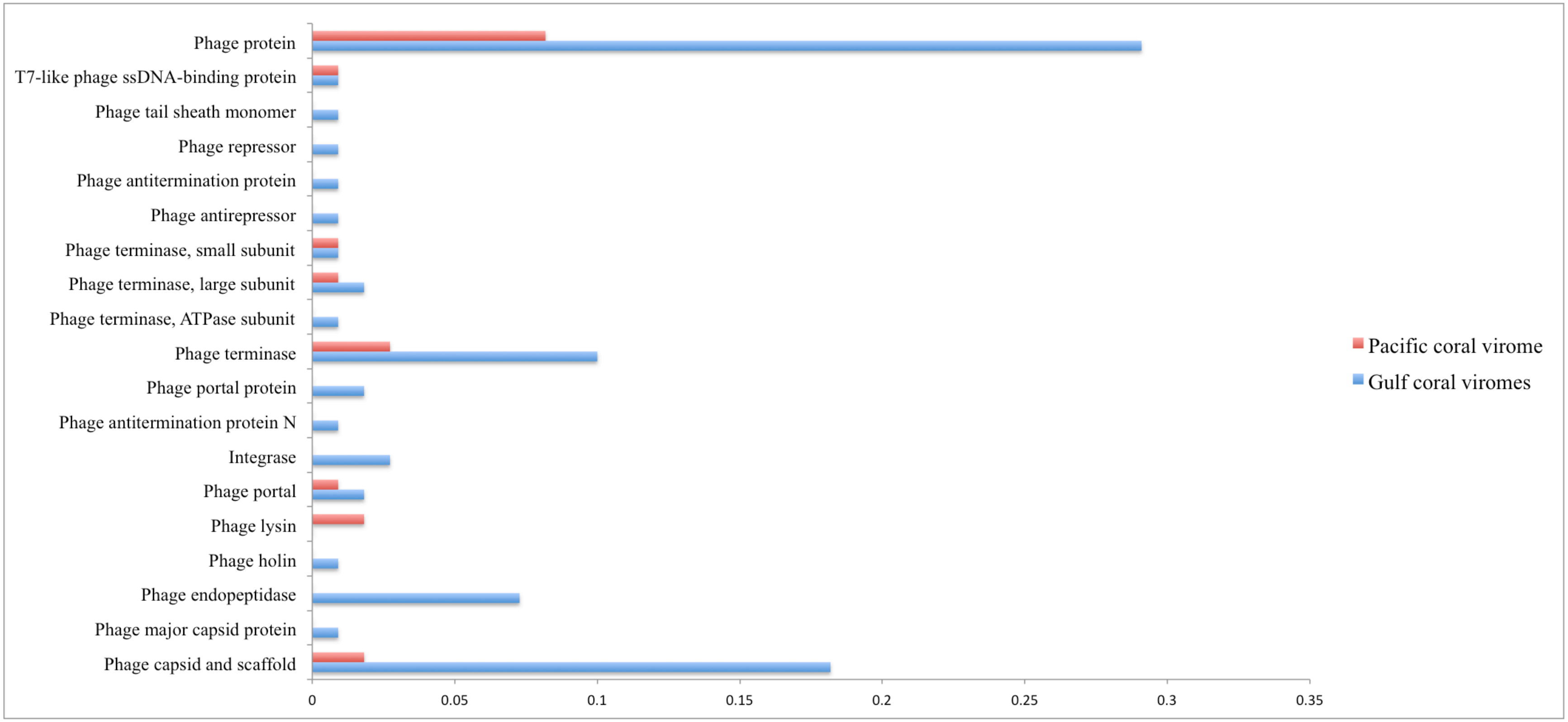

Supplement: Supplementary file 3 [file Image_1.PDF]
